# The Impact of the COVID-19 Pandemic: A Longitudinal Analysis of Body Weight Variations and Their Implications for Daily Habits

**DOI:** 10.3390/ijerph21111510

**Published:** 2024-11-13

**Authors:** Marina Martins Daniel, Juliana Costa Liboredo, Tamires Cássia de Melo Souza, Lucilene Rezende Anastácio, Alida Rosária Silva Ferreira, Lívia Garcia Ferreira

**Affiliations:** 1 Department of Nutrition, Universidade Federal de Lavras (UFLA), Lavras 37200-900, MG, Brazil; marinamardaniel@gmail.com; 2 Department of Nutrition, Universidade Federal de Minas Gerais (UFMG), Belo Horizonte 30130-1000, MG, Brazil; julianaliboredo@gmail.com; 3 Department of Food Science, Universidade Federal de Minas Gerais (UFMG), Belo Horizonte 31270-901, MG, Brazil; tamirescmsouza@gmail.com (T.C.d.M.S.); lucilene.rezende@gmail.com (L.R.A.); 4 Demography Post-Graduation Program, Economic Sciences, Universidade Federal de Minas Gerais (UFMG), Belo Horizonte 31270-901, MG, Brazil; alida.rsf@gmail.com

**Keywords:** body weight changes, pandemic, habits, behaviors

## Abstract

Assessing changes in lifestyle, dietary habits, eating behaviors, and stress during the pandemic and their impact on weight is crucial for developing effective interventions. This study investigated weight variations among Brazilians and associated habit changes over nine months during the COVID-19 pandemic. An online questionnaire was applied (T0/T1, T2). Weight variation classifications were determined from T0 to T1 and evaluated longitudinally for changes in lifestyle, dietary habits, food consumption, eating behavior, and perceived stress using generalized estimating equations (*p* < 0.05). Out of 453 participants, 23.6% lost weight, 18.1% maintained their weight, 26.0% gained up to 2.4 kg, and 32.2% gained ≥ 2.5 kg. Weight loss was associated with decreased food consumption and increased stress at T2. The group that gained up to 2.4 kg reported reduced food intake, snacking, meal preparation, and candy consumption but increased stress at T2. Those gaining ≥ 2.5 kg initially increased food consumption, snacking, meal preparation, hamburgers/canned products, sugary drinks, instant meals/snacks, candies, and fast food consumption at T1, all of which were reduced at T2, along with a decrease in uncontrolled and emotional eating. Although the pandemic initially affected daily habits differently based on weight changes, participants—especially those who gained more weight—tended to revert to pre-pandemic habits.

## 1. Introduction

The COVID-19 pandemic caused by the SARS-CoV-2 virus has significantly impacted the global population economically, politically, socially [1], and psychologically [2]. The first cases were recorded in December 2019, and the disease quickly spread from China to other countries, reaching Brazil in February 2020 [3]. By mid-2020, Brazil had implemented measures to contain the virus’s spread, such as social distancing and quarantining, that were strongly suggested in several places around the world [4]. This led to the closure of nonessential businesses, schools, and companies, with many employees transitioning to remote work. Initially, the population faced a challenging adjustment period, distancing from family and friends, altering daily routines, and grappling with the fear and uncertainty of the evolving pandemic [5].

By the end of 2020, with clearer and more consistent available information about the virus, the population adapted, authorities eased protective measures, and various activities resumed [6]. Brazil adopted measures to contain COVID-19 infections for an extended period, and in January 2021, a year after SARS-CoV-2 arrived in the country, vaccination began [3]. Despite the initiation of vaccinations in 2021, April of that year saw COVID-19 death tolls surpass those of 2020, making it the deadliest month of the pandemic in Brazil, with over 67,000 deaths [7].

Researchers observed unhealthy behaviors, including weight changes and depreobesity, related to the pandemic scenario in both overweight individuals and those with a normal body weight [8]. Sidor and Rzymski [9] noted that, while overweight individuals were likely to gain weight during quarantine, underweight individuals tended to lose weight. They concluded that the quarantine period accentuated pre-existing body weight changes and potential health issues related to weight. Additionally, social distancing contributed to weight gain during the first year of the COVID-19 pandemic, mainly due to increased screen time, reduced physical activity, and changes in behavior and food consumption [9,10,11,12,13,14,15]. Meta-analyses have shown that the pandemic’s onset altered weight and nutritional status [16,17], underscoring the importance of monitoring these changes over time. The need to determine whether individuals gained or lost weight at the pandemic’s start and to track these changes later became apparent. The continuous evaluation of these shifts is crucial, as they may relate to modifiable risk factors and inform strategic interventions for managing weight changes.

Given the aforementioned points, this study aimed to (1) identify the weight, BMI, and their classifications among Brazilian individuals during the pandemic; (2) classify weight variations in the initial months of the pandemic among these individuals; (3) investigate the variables influencing weight changes at the pandemic’s onset; and (4) track changes in lifestyle habits, eating behaviors, and perceived stress over 8–9 months, correlating these with observed weight changes during the pandemic.

## 2. Methods

This longitudinal study was conducted online via a questionnaire on the Google Forms^®^ platform. We included Brazilian volunteers aged 18 years or older, excluding pregnant women and those who submitted incomplete responses. The study relied on a convenience sample.

The study adhered to the Declaration of Helsinki and was approved by the Research Ethics Committee of the Federal University of Viçosa, Brazil (approval number 35516720.5.0000.5153). All participants provided informed consent through a free and informed consent form.

Participants were recruited using the snowball sampling technique [18]; they received the questionnaire link through social media, emails, and institutional channels and were encouraged to share it to broaden the participant base. The online semi-structured questionnaire was based on previous studies [9,13,19].

**Figure 1:**
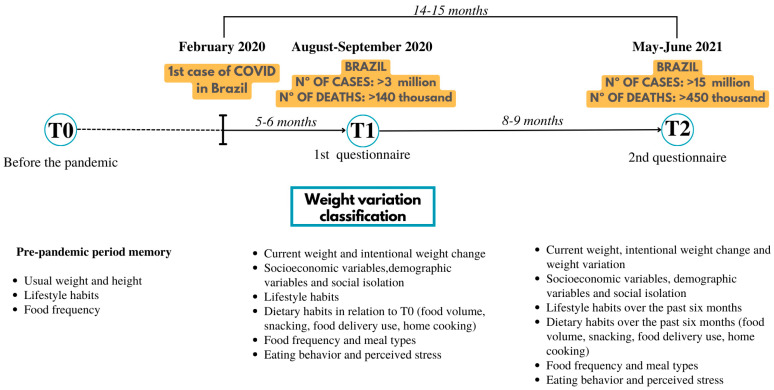
Variables collected from Brazilian individuals before the pandemic and during the pandemic at two time points.

The research consisted of two phases, as shown in Figure 1. Phase one occurred between August and September 2020, six months following the onset of the pandemic in Brazil. Participants answered single-choice questions related to weight variation, height, socioeconomic and demographic information, lifestyle, dietary habits, eating behaviors, and perceived stress at two intervals: before the pandemic (Time 0—T0) and during the study period (Time 1—T1). This phase established weight variation classifications and explored factors influencing these variations.

Phase two (Time 2—T2) occurred 14–15 months later, during Brazil’s second COVID-19 peak in May–June 2021. Participants from the first phase completed the questionnaire again via email. This phase aimed to track changes in lifestyle habits, dietary patterns, eating behavior, and perceived stress, correlating these with the weight classifications identified in phase one. The study concluded by evaluating changes in weight and nutritional status from T0 to T2.

Retrospective pre-pandemic data were collected for T0, while data for T1 and T2 were collected during the study period. On average, participants spent about 15 min completing the questionnaires.

### 2.1. Variation in Weight and Nutritional Status

The questions “What was your usual weight prior to the pandemic?” and “How much do you think you weigh now?” were used in the questionnaire to determine the participants’ usual weight (T0) and current weight (T1), respectively. These questions were adapted from the Vigitel (Sistema de Vigilância de Fatores de Risco e Proteção para Doenças Crônicas por Inquérito Telefônico) [20], a telephone survey conducted by the Brazilian Ministry of Health in all state capitals and the Federal District to retrieve data from a sample of the adult population.

Weight variation classifications were determined using participants’ usual weight at T0 compared to their current weight at T1. The difference between T1 and T0 was used to categorize participants into four groups: “lost“(≤−0.5 kg), “maintained“ (−0.5 kg to 0.5 kg), “gained up to 2.4 kg”, and “gained ≥ 2.5 kg” according to Bhutani et al. [10]. Conversions from pounds to kilograms were rounded to the nearest whole number. Additionally, participants were queried regarding the intentionality of their weight change.

Body mass index (BMI) was calculated at T0, T1, and T2 using reported body weight and height to determine weight status at various time points throughout the study. BMI categories adhered to WHO standards [21].

### 2.2. Lifestyle Habits

Participants reported their screen time across devices (hours/day), alcohol consumption frequency (times/week) and amount (dosage/occasion), physical activity (times/week), sleep duration (hours/day), sleep quality (unchanged, better, or worse), and cigarette usage (number/day) before the pandemic (T0) and at the time they were filling out the questionnaires during the pandemic (at T1 and T2). These data were coded following protocols from a prior study [22].

### 2.3. Dietary Habits

Participants detailed changes in food consumption volume, snacking habits, use of food delivery services, and cooking practices at home in the last six months. They also reported meal types, including breakfast, morning snacks, lunch, afternoon snacks, dinner, evening snacks, and other meals during the questionnaire period. Food consumption frequency (before the pandemic—T0—and at the time they were filling out the questionnaires during the pandemic—at T1 and T2) was evaluated using a food frequency questionnaire adapted from the Food and Nutrition Surveillance System (SISVAN) [23]. Reports included consumption of legumes, vegetables, fresh fruits, cereals, meats, milk and dairy, bakery items, canned products, hamburgers, sugary drinks, instant meals and snacks, candies, and fast food. Consumption of the last five foods listed were considered ultra-processed in this study, according to the NOVA classification [24]. Coding for dietary variables and meal timing followed the standards outlined in a previous study [22].

### 2.4. Eating Behavior

Eating behaviors were assessed using the Three-Factor Eating Questionnaire (TFEQ-R21), validated in Brazil by [25]. This tool measures cognitive restriction, uncontrolled eating, and emotional eating through 21 questions. Participants were asked to answer about their eating behavior at the time of the survey (T1 and T2). Questions 1–20 include 4 answer possibilities (which vary according to the question) and question 21 is answered using a numerical scale from 1 to 8.

### 2.5. Perceived Stress

Perceived stress was measured using the Perceived Stress Scale (PSS-10), which was translated and validated for Brazilian adults by [26]. This scale includes 10 items rated on a five-point Likert scale, assessing stress over the past 30 days. Items are reverse-scored appropriately, with total scores ranging from 0 to 40 and higher scores signifying greater stress [26].

### 2.6. Data Analysis

Data analysis was conducted using the Statistical Package for the Social Sciences (SPSS Inc., Chicago, IL, USA), version 25.0. Results are reported as medians and interquartile ranges for non-normal distributions (Shapiro–Wilk test; *p* < 0.05) or as frequencies and absolute numbers. Changes in weight, BMI, and nutritional status between T0, T1, and T2 were evaluated using the Friedman test with a Bonferroni correction for paired numerical variables. The Cochran’s Q test, also with a Bonferroni correction, was applied to categorical variables.

Variables related to the weight variation classifications “lost”, “maintained”, “gained up to 2.4 kg”, and “gained ≥ 2.5 kg” and their changes over time were analyzed using generalized estimating equations (GEEs). The GEEs, employing a log-link function, facilitated both between-group and within-group comparisons. Model selection was determined by the lowest quasi-likelihood under the independence model criterion (QIC) value.

Statistical significance was established at *p* < 0.05 for all tests. Box plots were utilized for visualizing data where significant differences were identified, especially when medians and quartiles did not clearly depict the highest or lowest values. The effect sizes (d) were calculated by G*Power software (version 3.7.1), and were considered the following cutoffs: d < 0.19: insignificant; d = 0.2 to 0.49: small; d = 0.5 to 0.79: medium; and d > 0.8: large [27].

## 3. Results

**Figure 2:**
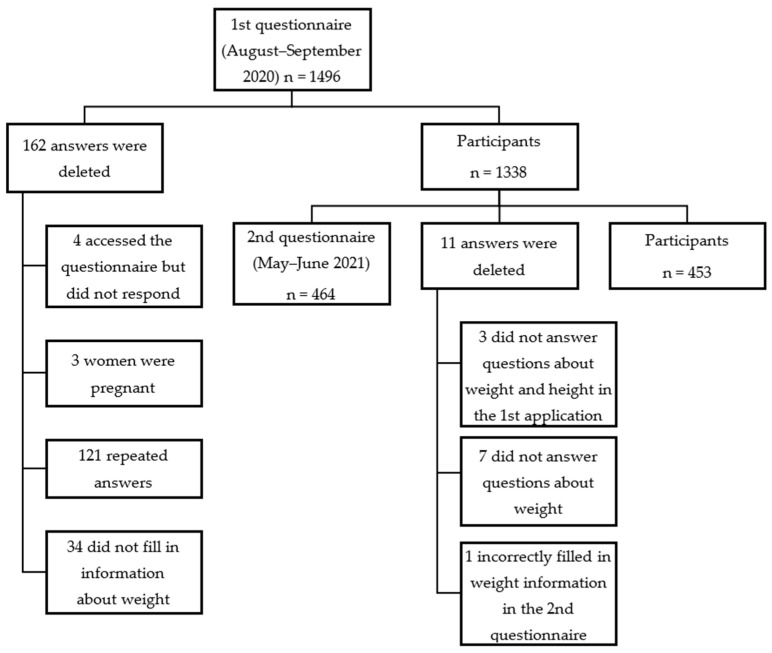
Flowchart of study participants.

In the first phase (T1) of the study, 1334 individuals completed the questionnaire. The second phase (T2) saw 464 respondents, with 11 excluded, resulting in a total of 453 participants, as shown in Figure 2.

Participants had a median age of 32 years (range 24–40) in both phases. Females comprised 83% (n = 376) of the cohort. Most participants (79.9%) lived in the southeast region, 42.6% of whom reported having complete or incomplete graduate degrees and family incomes ranging from R$4180.00 to R$5225.00. At T1, 36.9% (n = 167) lived with their parents, decreasing slightly to 34.2% (n = 155) at T2 (*p* < 0.05). Initially, 60.3% (n = 273) adhered to total social isolation, which reduced to 45.3% (n = 205) by T2 (*p* < 0.001).

### 3.1. Variations in Weight, BMI, and Weight Change Classification During the Pandemic

**Figure 3:**
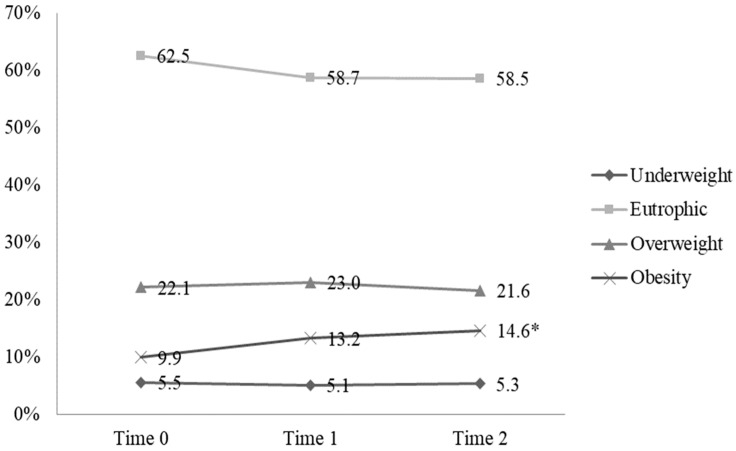
BMI classification of study participants at T0, T1, and T2. Cochran’s Q test (*p* = 0.002). * Cochran’s Q test (*p* = 0.002).

We identified a significant weight and BMI increase between T0 and T1, with no substantial changes between T1 and T2 (Table 1). Obesity rates increased significantly from T0 to T1 and from T0 to T2 (*p* < 0.05), marking a 4.7% rise from the pre-pandemic period (Figure 3). Most participants did not intentionally create these weight changes (67.8% from T0 to T1 and 66.7% from T1 to T2).

Regarding weight variations, 23.6% (n = 107) lost weight, 18.1% (n = 82) maintained their weight, and 58.2% experienced a weight gain between T0 and T1, with 26.0% (n = 118) gaining up to 2.4 kg and 32.2% (n = 146) gaining ≥ 2.5 kg. Notably, among those overweight or obese before the pandemic, 38.0% (n = 38) and 53.3% (n = 24), respectively, gained ≥ 2.5 kg, while 31.0% (n = 31) and 26.7% (n = 12) of the same groups lost weight at T1. Additionally, 52% (n = 13) of those underweight at T0 gained weight (16.0% (n = 4) up to 2.4 kg and 36.0% (n = 9) ≥ 2.5 kg).

Table 2 displays the median weights of participants at T0, T1, and T2, categorized by weight change group. We observed that participants with higher initial weights at T0 either lost weight or gained ≥ 2.5 kg [row analyses]. The data reveal that those who lost weight at T1 continued this trend at T2 (*p* < 0.001). In contrast, participants who gained weight at T1 exhibited no significant weight changes at T2 [column analysis].

### 3.2. Influence of Variables on Weight Variations at the Pandemic’s Onset

Table 3 outlines the significant associations between lifestyle, dietary habits, and eating behaviors, with weight classification changes from T0 to T1. The effect size was between 0.5 and 0.79 for all results, except for physical activity (d = 0.36) and the consumption of hamburgers and canned products (d = 0.83). The complete analyses are detailed in Supplementary Tables S1–S5 [row analyses].

Participants who gained weight reported a higher frequency and dosage of alcohol consumption compared to those who maintained their weight (*p* < 0.001). Those who lost weight engaged in 40 min more of physical activity on average than those in the “gained ≥ 2.5 kg” group.

Changes in food consumption volume and snacking behavior had significant associations with weight classifications. The “gained ≥ 2.5 kg” group increased their food consumption and snacking at T1 compared to the “lost” and “maintained” groups (*p* < 0.05). Conversely, most of the “lost” group either reduced or maintained their food intake and snacking habits (*p* < 0.05). The frequency of daily meal adherence showed no differences among the groups (*p* > 0.05).

Distinct patterns emerged in the consumption frequency of specific foods across the groups. The “gained ≥ 2.5 kg” group consumed more hamburgers or canned products, instant meals and snacks, and candies compared to the “lost” and “maintained” groups (*p* < 0.05). This group also had a higher intake of sugary drinks and fast food (*p* < 0.05). There was also a noticeable reduction in fresh fruit consumption in this group compared to the “lost” and “gained up to 2.4 kg” groups (*p* < 0.05).

Eating behaviors and perceived stress levels also varied by weight change group. Participants who gained ≥ 2.5 kg exhibited higher levels of uncontrolled and emotional eating (*p* < 0.05). This group also reported higher perceived stress levels (*p* = 0.004) compared to the “maintained” and “gained up to 2.4 kg” groups, but similar to the group that lost weight.

### 3.3. Changes in Lifestyle and Dietary Habits, Eating Behavior, and Perceived Stress over Eight to Nine Months During the Pandemic Based on Body Weight Variations

**Figure 4:**
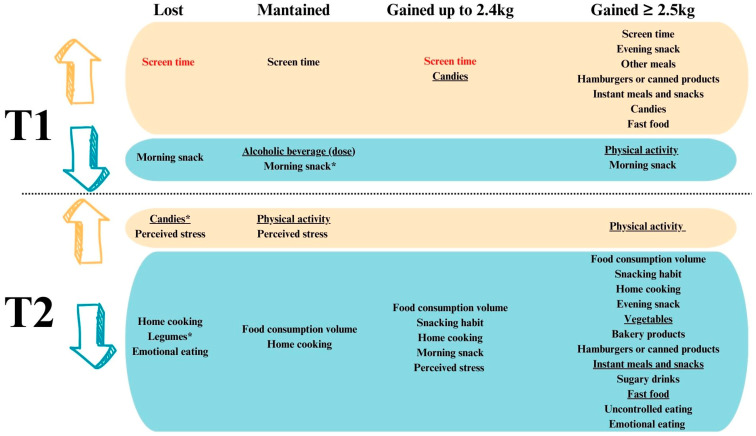
Statistically significant changes in lifestyle and dietary habits, eating behavior, and perceived stress over eight to nine months during the pandemic (T0–T1; T1–T2; T0–T2) with each weight change group. * in relation to T0. The effect sizes were as follows: between 0.2 and 0.49 for the underlined variables, above 0.8 for the variables in red, and between 0.5 and 0.79 for the remaining variables.

Figure 4 presents the results of statistically significant changes in lifestyle and dietary habits, eating behavior, and perceived stress, assessed longitudinally (T0–T1; T1–T2; T0–T2). All complete analyses and their respective values of effect size are displayed in Supplementary Tables S1–S5 [column analysis]. We observed a consistent increase in screen time across all groups from T0 to T1, which remained elevated at T2. Among participants who maintained their weight, the amount of alcohol consumed was significantly lower (*p* = 0.033) between T0 and T1. Additionally, this group exhibited an increase in physical activity during the pandemic (T1–T2). Conversely, the group that gained ≥ 2.5 kg decreased physical activity from T0 to T1 but showed a significant increase from T1 to T2 (*p* < 0.001).

During the follow-up period (T1–T2), both the frequency of increased food volume and snacking declined at T2 in groups that gained weight (*p* < 0.05). Notably, in the group that gained ≥ 2.5 kg, there was an increase in participants who abstained from snacking at T2 (*p* < 0.05). A widespread increase in home meal preparation was noted at T1 across all groups, followed by a significant decrease at T2 (*p* < 0.05). The usage of food delivery services surged by over 45% at T1 and 37% at T2 in all groups, with no significant differences in usage rates.

In terms of meal consumption, the number of participants consuming morning snacks decreased from T0 to T1 in the “lost”, “maintained”, and “gained ≥ 2.5 kg” groups and from T0 to T2 in the “gained up to 2.4 kg” group (*p* < 0.05). The “gained ≥ 2.5 kg” group alone reported an increase in the consumption of evening snacks and other meals from T0 to T1, with a significant reduction in evening snack consumption at T2 (*p* < 0.05).

Analysis of food consumption frequency throughout the study revealed no change in the “lost” group from T0 to T1 and T1 to T2. From the pre-pandemic period (T0) to the second time point (T2), there was a reduction in legume consumption and an increase in candy consumption (*p* < 0.05). The “gained ≥ 2.5 kg” group reported increased consumption of hamburgers or canned products, instant meals and snacks, candies, and fast food from T0 to T1. However, there was a decline in the consumption of vegetables, bakery products, hamburgers or canned products, sugary drinks, instant meals and snacks, candies, and fast food from T1 to T2 among participants in this group (*p* < 0.05).

Finally, between T1 and T2, there was an increase in perceived stress in the “lost”, “maintained”, and “gained up to 2.4 kg” groups. Additionally, there was a reduction in emotional and uncontrolled eating in the “lost” and “gained ≥ 2.5 kg” groups.

## 4. Discussion

This study analyzed weight variation classifications during the initial phase of the pandemic and examined how participants altered their lifestyle and dietary habits in response to changes in their weight profile. As evidenced by previous research, a notable consequence of the COVID-19 pandemic was an increase in population weight gain [9,28].This trend continued in our study, with 58.2% of participants gaining weight and a 4.7% rise in obesity rates from the pre-pandemic period (T0) to the second questionnaire’s administration (T2). By comparison, studies conducted in the Greater Middle East [29], Northern Italy [30], and Spain [31] reported lower weight gain percentages of 30.3%, 39.0%, and 38.8%, respectively. This higher weight gain in Brazilians is particularly concerning given that, even prior to the pandemic, over half of Brazilians were overweight [20]. The data from this study followed the trend of increasing excess weight in the Brazilian population at the beginning of the pandemic, as national survey data already showed an increase of approximately 2.1% in data collected between January and April 2021 [32].

Furthermore, individuals who were already overweight or obese at the onset of the pandemic were more likely to gain ≥ 2.5 kg. Sideli et al. [17] conducted a meta-analysis of studies from January 2020 to January 2021 on individuals with obesity and eating disorders, finding that 50% of individuals with obesity experienced weight gain during the pandemic.

In addition to weight variations, changes in lifestyle and dietary habits were initiated at the onset of the pandemic (T1). Individuals who gained weight reported significant increases in food consumption volume, snacking habits, and intake of hamburgers or canned products, instant meals and snacks, sugary drinks, and fast food. In contrast, their consumption of fresh fruits decreased. This aligns with findings by Lim et al. [33], who noted that pandemic-induced dietary changes were directly associated with an increased risk of weight gain and subsequent obesity. These dietary shifts primarily involved greater reliance on convenient takeaway foods, and a corresponding decrease in the consumption of healthy, homemade meals [33].

These changes in dietary habits, characterized by increased consumption of ultra-processed foods, may be attributed to the social and economic impacts of the pandemic. Isolation and lockdown measures led to financial constraints, influencing food choices and favoring low-quality, high-calorie foods rich in sodium, trans fats, saturated fats, and sugars. These foods are often more affordable [34]. Additionally, the accessibility of such foods increased through delivery apps, with a notable 20–25% rise in usage during the 2020 pandemic in Brazil [35]. Our study observed this trend across all groups with varying weight changes. Moreover, the consumption of chocolates, sweets, pastries, snacks, and instant appetizers likely increased as these “comfort foods” can temporarily enhance mood following intake [36]. Research indicates a surge in the consumption of these foods during periods of social isolation [37,38].

Our study found that the “gained ≥ 2.5 kg” group scored higher in uncontrolled eating and emotional eating compared to all other groups, and at the initial research stage (T1), scored higher in perceived stress compared to the “maintained” and “gained weight up to 2.4 kg” groups, corroborating the weight variation classification findings. According to Arora et al. [39], negative emotions, exacerbated by the pandemic, correlate with food intake and an individual’s susceptibility to these emotions or challenging situations. These emotions significantly influence food choices, as certain foods can alter emotional states through neurotransmitter release, which regulates mood, body temperature, and blood stability [40]. Studies suggest that individuals prone to emotional eating were more likely to overeat in response to negative emotions during the pandemic [22], seek comfort in food due to stress, and increase their intake of highly palatable, high-calorie foods [41]. However, this is a new area of study that is still very exploratory and theoretical.

During the pandemic, groups that gained weight reported a higher frequency and greater amount of alcohol consumption compared to the “maintained” group. Increased alcohol intake was also reported as a compensatory mechanism for caloric intake to inhibit appetite, lose weight, or prevent weight gain [42], likely as a response to psychological distress caused by social isolation and the ongoing uncertainty [43]. Physical activity played a significant role in weight fluctuation; participants who lost weight from T0 to T1 increased their weekly physical activity by 40 min. This behavior was essential for maintaining immunity, improving cardiometabolic health, and enhancing mental well-being during the pandemic [44]. A systematic review on pandemic-induced social isolation and body weight showed that individuals incorporating physical activity and a diet rich in fruits, vegetables, and legumes were more likely to lose weight [45]. Furthermore, our study observed that the group that lost weight consumed more fruits than the group that gained at least 2.5 kg.

In the pandemic’s second phase, the weight gain groups maintained their weight, contrasting with Bhutani et al. [10], who reported ongoing weight gain from the first (April/May 2020) to the second evaluation period (September/October 2020). This stabilization in weight could be attributed to the increased physical activity and dietary modifications seen between T1 and T2. Specifically, the “gained ≥ 2.5 kg” group decreased their intake of high-calorie and nutritionally inadequate foods by T2, alongside a reduction in food consumption and snacking. Bull et al. [46] suggested that these changes or the re-adoption of habits could be related to the perceptions of weight gain and the desire to lose/restore weight. This group also reduced their bakery product consumption at T2, possibly because of the recognized link between refined bread and excess abdominal fat [47]. Additionally, they reduced their evening snack consumption by T2; reducing evening snacks is beneficial, as they have been associated with weight gain during the pandemic [12]. Furthermore, the decrease in food consumption and snacking between T1 and T2 in the “gained ≥ 2.5 kg” group may reflect the easing of strict social isolation measures that occurred in the second phase of research. Possibly, the decline in emotional and uncontrolled eating at T2 contributed to these dietary and lifestyle changes. The impact of eating behavior on food intake is a crucial aspect of this discussion.

Key findings from our study highlight the “lost” group, which demonstrated continuous weight loss from T0 to T1 and T1 to T2. Despite high stress levels, they maintained physical activity levels and reduced emotional eating, corroborating findings by Bhutani et al. [10], that may have contributed to weight loss.

We also observed an initial increase in home cooking at T1 across all weight groups, which decreased by T2. Initially, social isolation likely encouraged more cooking at home, but as restrictions eased, this practice declined [34]. Concurrently, screen time increased in all weight groups and remained high into T2. Although essential for communication during quarantine, increased screen use has been associated with poorer dietary choices and higher consumption of ultra-processed foods [11,48]. Additionally, the reduction in morning snack consumption is concerning, as such snacks are inversely related to obesity due to their lower energy density [49].

During the research’s initial phase, the population was adapting to a scenario filled with uncertainties, which often induced stress and impacted food choices, leading to weight gain [12]. By T2, with the advent of vaccination and the resumption of normal activities by several institutions, the population had adapted to the ongoing situation despite a rising death toll. In this study, during the second phase, there was a reduction of 15% (from 60.3% to 45.3%) in social isolation among participants. These conditions may have facilitated the recovery of dietary habits observed from T1 to T2, as well as weight gain amid the pandemic, suggesting that the initial adverse effects of the pandemic on habits and quality of life can gradually improve with new perspectives on a routine that is closer to normal [11,50].

The present study has limitations and strengths. Firstly, it includes a sample that may not be representative, as most participants were female (a similar finding in previous research conducted during the pandemic [9,13,51]), predominantly eutrophic, and from the southeast region of Brazil. The use of an online questionnaire restricted participation to those with internet access. Self-reported data (such as current weight), as well as the use of retrospective weight, may have led to overestimations or underestimations; we do not rule out the possibility that normal weight fluctuations may be present, such as those related to the female menstrual cycle. Furthermore, data on weight and eating practices from T0 were retrospective, relying on the participants’ memories, which may have influenced the results of the comparison of the time interval T0–T1 [9,15,52].

Although sample losses are expected in longitudinal studies, another limitation was the considerable decrease in the number of participants during the study. Possibly, this reduction occurred due to the study’s online design, which required participants to be available to access and answer the questionnaire alone more than once. Since the COVID-19 vaccine had already begun and numerous activities and face-to-face work had resumed in T2, participants may have been less willing to participate in this phase of the survey. Sample attrition occurred between the first and second assessments (n = 881) but did not bias results, as weight variation classifications at T1 showed no significant differences (*p* = 0.105) between non-respondents at T2 and participants in the second phase (Supplementary Table S6).

Nonetheless, this study stands out as one of the few to assess participants longitudinally during Brazil’s strictest social isolation measures of the COVID-19 emergency. It contributes valuable insights into the associations between body weight variations and habits and behaviors in response to different pandemic scenarios within the country. Our significant results do not have any insignificant effect sizes, and most of the effect sizes are between 0.49 and 0.79, indicating a medium effect and consistent findings.

Furthermore, the study’s findings are critical for understanding changes in lifestyle, dietary habits, and eating behaviors that could have damaged the population’s health during this abnormal period. Thus, this study is extremely necessary, particularly in Brazil, a nation heavily impacted by COVID-19 and with high rates of overweight. We can interpret the study’s conclusions as potential shifts that could occur during future crises or periods of social turmoil. Additionally, we can use them to develop multidisciplinary health interventions in these situations.

## 5. Conclusions

More than half of the participants gained weight during the pandemic, with the majority falling into the “gained ≥ 2.5 kg” category. Those who lost weight during this period maintained healthier lifestyle and dietary habits compared to those who gained weight, reinforcing the later observed weight loss trend. The “gained ≥ 2.5 kg” group decreased their physical activity while increasing their food consumption, snacking, and intake of ultra-processed foods. Despite these trends, a return to healthy dietary and lifestyle habits emerged, marked by a reduction in emotional and uncontrolled eating during the pandemic.

## Figures and Tables

**Table 1:** Weight and body mass index variations in the participants at the three study time points (T0, T1, and T2).

	T0 % (n)	T1 % (n)	T2 % (n)	*p*
Weight ^1^	63.0 (55.0–72.0) ^a^	64.0 (56.0–75.0) ^b^	64.0 (56.0–75.0) ^b^	<0.001
BMI (kg/m^2^) ^1^	22.9 (20.9–26.0) ^a^	23.5 (21.0–26.5) ^b^	23.5 (21.0–26.9) ^b^	<0.001
Weight variation (kg) ^2^	1.0 (0.0–3.0)		-	<0.001
	-	0.0 (−1.95–2.0)		

Legend: BMI: body mass index; ^1^ Friedman test with a Bonferroni adjustment (^a, b^ different letters indicate significant changes); ^2^ Friedman test.

**Table 2:** Weight medians of the participants at the three study time points (T0, T1, and T2) in each weight change group.

		Weight Variation Groups				
Item	Time	Lost (n =107)	Maintained (n = 82)	Gained up to 2.4 kg (n = 118)	Gained ≥ 2.5 kg (n = 146)	*p*
Weight (kg)	T0	63.0 ^a1^ (55.0–75.0)	60.0 ^2^ (53.0–71.2)	59.0 ^a2^ (54.0–66.0)	67.0 ^a1^ (60.0–78.0)	
	T1	62.00 ^b^ (52.0–71.0)	60.0 (53.0–71.2)	61.0 ^b^ (55.0–67.2)	72.0 ^b^ (63.7–81.2)	<0.001
	T2	60.0 ^c^ (53.0–75.0)	61.0 (53.9–71.6)	60.0 ^b^ (54.0–69.0)	71.0 ^b^ (62.3–83.0)	
	*p*	<0.001	0.098	<0.001	<0.001	

Generalized estimating equations for between-group (^1, 2^ different numbers indicate significant changes) and within-group analyses (^a, b, c^ different letters indicate significant changes).

**Table 3:** Statistically significant associations between lifestyle, dietary habits, eating behavior and perceived stress of the participants with each weight change group from T0 to T1.

	Lost (n = 107)	Maintained (n = 82)	Gained up to 2.4 kg (n = 118)	Gained ≥ 2.5 kg (n = 146)	*p*
**Lifestyle habits**					
Frequency of alcoholic beverage intake (times/week)	0.5 (0–1) ^1,2^	0.5 (0–0.5) ^2^	1.0 (0.5–2.5) ^1^	0.5 (0–2.5) ^1^	<0.001 (d = 0.54)
Alcoholic beverages (dosage/occasion)	1.0 (0–2.5) ^1,2^	1.0 (0–2.1) ^2^	1.0 (1–2.5) ^1^	2.5 (0–4.5) ^1^	<0.001 (d = 0.68)
Physical activity (minutes/week)	120 (0–180) ^1^	80 (0–150) ^1,2^	80 (0–120) ^1,2^	80 (0–120) ^2^	0.002 (d = 0.41)
**Dietary habits**					
Food consumption volume (%, n)					
Increased	27.1 (29) ^1^	43.9 (36) ^1,2^	55.9 (66) ^2^	82.2 (120) ^3^	<0.001 (d = 0.65)
Decreased	39.3 (42) ^1^	9.8 (8) ^2^	8.5 (10) ^2^	9.6 (14) ^2^	<0.001 (d = 0.61)
Remained the same	31.8 (34) ^1^	43.9 (36) ^1^	32.2 (38) ^1^	6.8 (10) ^2^	<0.001 (d = 0.73)
Snacking habits (%, n)					
Increased	27.1 (29) ^1^	32.9 (27) ^1^	52.5 (62) ^2^	67.8 (99) ^2^	<0.001 (d = 0.54)
Decreased	17.8 (19) ^1^	6.1 (5) ^1,2^	5.9 (7) ^2^	3.4 (5) ^2^	<0.001 (d = 0.57)
Does not snack	30.8 (33) ^1^	29.3 (24) ^1^	19.5 (23) ^1,2^	11.0 (16) ^2^	<0.001 (d = 0.60)
Frequency of consumption (days/week)					
Fresh fruits	7.0 (5.0–10.0) ^1^	7.0 (5–10) ^1,2^	7.0 (4.3–10) ^1^	6.0 (2.5–10) ^2^	0.007 (d = 0.52)
Hamburgers or canned products	0.5 (0.5–2.5) ^1^	0.5 (0.5–1.0) ^1^	1.0 (0.5–2.5) ^1,2^	2.5 (0.5–5.0) ^2^	0.001 (d = 0.83)
Sugary drinks	0.5 (0.5–2.5) ^1^	0.5 (0.5–2.5) ^1^	0.5 (0.5–2.5) ^1^	1.0 (0.5–5) ^2^	0.011 (d = 0.61)
Instant meals and snacks	0.5 (0.5–1) ^1^	0.5 (0–1) ^1^	0.5 (0.5–1) ^1,2^	1.0 (0.5–2.5) ^2^	<0.001 (d = 0.54)
Candies	1.0 (0.5–5) ^1^	2.5 (0.5–5) ^1^	2.5 (1–7) ^1,2^	5.0 (1–7) ^2^	0.002 (d = 0.64)
Fast food	1 (0.5–1) ^1^	1 (0.5–1) ^1^	1 (0.5–2.5) ^1^	1 (1–2.5) ^2^	<0.001 (d = 0.43)
**Eating behaviors**					
Uncontrolled eating	25.9 (14.8–40.7) ^1^	20.3 (8.3–36.1) ^1^	25.9 (16.6–44.4) ^1^	40.7 (22.2–55.5) ^2^	<0.001 (d = 0.73)
Emotional eating	27.7 (5.5–50.0) ^1^	27.7 (5.5–38.8) ^1^	27.7 (11.1–50.0) ^1^	50.0 (22.2–72.2) ^2^	<0.001 (d = 0.70)
**Perceived stress**	23.0 (19.0–27.0) ^1,2^	20.0 (15.2–27.7) ^1^	22.0 (16.0–27.5) ^1^	25.0 (21–30.0) ^2^	0.004 (d = 0.60)

Generalized estimating equations for between-group analyses (^1, 2, 3^ different numbers indicate significant changes).

## Data Availability

The datasets presented in this article are not readily available for ethical reasons. Requests for access to datasets should be directed to livia.ferreira@ufla.br.

## Supplementary Materials

The following supporting information can be downloaded at: https://www.mdpi.com/article/10.3390/ijerph21111510/s1, Table S1: Lifestyle habits of study participants at T0, T1, and T2 for each weight variation group; Table S2: Dietary habits of study participants at T1 and T2 for each weight variation group; Table S3: Types of meals consumed by study participants at T0, T1, and T2 for each weight variation group; Table S4: Frequency of food consumption by study participants at T0, T1, and T2 for each weight variation group; Table S5: Eating behavior and perceived stress of study participants at T1 and T2 for each weight variation group; Table S6: Classification of weight variation of participants in T1, according to participation in the second phase (T2) of the study.
