# Supplementary material for: The Impact of the COVID-19 Pandemic: A Longitudinal Analysis of Body Weight Variations and Their Implications for Daily Habits
Source: Int J Environ Res Public Health. 2024 Nov 13;21(11):1510. doi: 10.3390/ijerph21111510 (PMC11594231; doi:10.3390/ijerph21111510)
Supplement: Supplementary file 1 [file ijerph-21-01510-s001.zip › ijerph-3209841-supplementary.pdf]

Supplementary Materials

Table S1 – Lifestyle habits of study participants at T0, T1, and T2 for each weight variation group

	**T**	**Lost (n=107)**	**Maintained (n=82)**	**Gained up to 2.4kg (n=118)**	**Gained ≥ 2.5kg (n=146)**	**p***
Screen time (hours/day)	**T0**	6.5[a] (3.0 - 6.5)	6.5[a] (3.0 - 6.5)	6.5[a] (3.0 - 6.5)	6.5[a] (3.0 - 6.5)	
	**T1**	10.5[b] (6.5 - 14.5)	10.5[b] (6.5 - 11.5)	10.5[b] (6.5 - 14.5)	10.5[b] (6.5 - 14.5)	0.388 (d = 0,09)
	**T2**	10.5[b] (6.5 - 10.5)	6.5[b] (6.5 - 10.5)	10.5[b] (6.5 - 10.5)	10.5[b] (6.5 - 10.5)	
**p****		<0.001 (d = 85)	<0.001 (d = 0,73)	<0.001 (d = 0,84)	<0.001 (d = 0,78)	
Frequency of alcoholic beverage intake (times/week)	**T0**	0.5 (0 - 2.5)	0.5 (0 - 1.0)	1 (0.5 - 2.5)	0.5 (0 - 2.5)	
	**T1**	0.5 (0 – 1.0)[1,2]	0.5 (0 - 0.5)[2]	1.0 (0.5 - 2.5)[1]	0.5 (0 - 2.5)[1]	<0.001 (d = 0,30)
	**T2**	0.5 (0 – 1.0)	0.5 (0 - 0.6)	1.0 (0.5 - 2.5)	0.5 (0 – 1.0)	
**p****		0.118 (d = 0,11)	0.056 (d = 0,15)	0.397 (d = 0,07)	0.80 (d = 0,06)	
Alcoholic beverage (dose/occasion)	**T0**	2.5 (0 - 4.5)	1.0[a] (0 - 2.5)	2.5 (1- 2.5)	2.5 (0.2 -2.5)	
	**T1**	1.0 (0 - 2.5)[1,2]	1.0[b] (0 - 2.1)[2]	1.0 (1 - 2.5)[1]	2.5 (0 - 4.5)[1]	<0.001 (d = 0,36)
	**T2**	1.0 (0 - 2.5)	1.0[ab] (0 - 2.5)	1.0 (1.0 - 2.5)	2.5 (1.0 - 2.5)	
**p****		0.167 (d = 0,12)	0.033 (d = 0,24)	0.619 (d = 0,09)	0.517 (d = 0,11)	
Physical activity (minutes/week)	**T0**	120 (80 – 240)	120[ab] (0 – 240)	120 (80 – 180)	120[a] (0 – 180)	
	**T1**	120 (0 – 180)[1]	80[a] (0 – 150)[1,2]	80 (0 – 120)[1,2]	80[b] (0 -120)[2]	0.002 (d = 0,41)
	**T2**	120 (0 – 180)	120[b] (80 – 240)	120 (80 – 180)	80[c] (0 – 120)	
**p****		0.082 (d = 0,17)	0.018 (d = 0,35)	0.079 (d = 0,17)	<0.001 (d = 0,36)	
Sleeping time (hours/day)	**T0**	8.0 (7.5 – 9.0)	8.0 (7.0 – 9.0)	8.0 (7.0 - 8.7)	8.0 (7.0 – 9.0)	
	**T1**	8.3 (7.5 – 9.0)	8.0 (7.0 – 9.0)	8.0 (7.4 – 9.0)	8.0 (7.0 – 9.0)	0.568 (d = 0,1)
	**T2**	8.0 (7.1 – 8.5)	8.0 (7.4 – 8.5)	8.0 (7.0 – 8.5)	8.0 (7.0 – 8.5)	
**p****		0.853 (d = 0,08)	0.218 (d = 0,14)	0.593 (d = 0,11)	0.270 (d = 0,10)	

Generalized Estimating Equations *between-group (different numbers indicate significant changes) and **within-group analyses (different letters indicate significant changes)

Note: There was no improvement and/or worsening in sleep quality in relation to weight variation during the two periods of the pandemic (p=0.750; p=0.812). Cigarette consumption was low, as the median and quartiles were always equal to 0.00 (0.00 – 0.00).

Table S2 – Dietary habits of study participants at T1 and T2 for each weight variation group

	T	Lost (n=107) % (n)	Maintained (n=82) % (n)	Gained up to 2.4kg (n=118) % (n)	Gained ≥ 2.5kg (n=146) % (n)	p*
**Food consumption volume**						
Increased	**T1**	27.1 (29)[1]	43.9 (36)[1,2]	55.9 (66)[2]	82.2 (120)[3]	<0.001 (d = 0,59)
	**T2**	29.0 (31)	26.8 (22)	38.1 (45)	45.2 (66)	
**p****		0.746 (d = 0,08)	0.008 (d = 0,52)	0.004 (d = 0,64)	<0.001 (d = 0,72)	
Decreased	**T1**	39.3 (42)[1]	9.8 (8)[2]	8.5 (10)[2]	9.6 (14)[2]	<0.001 (d = 0,61)
	**T2**	29.9 (32)	15.9 (13)	18.6 (22)	21.9 (32)	
**p****		0.086 (d = 0,29)	0.225 (d = 0,11)	0.023 (d = 0,52)	0.002 (d = 0,78)	
Remained the same	**T1**	31.8 (34)[1]	43.9 (36)[1]	32.2 (38)[1]	6.8 (10)[2]	<0.001 (d = 0,58)
	**T2**	40.2 (43)	57.3 (47)	43.2 (51)	32.9 (48)	
**p****		0.139 (d = 0,12)	0.063 (d = 0,21)	0.037 (d = 0,48)	<0.001 (d = 0,65)	
**Snacking habit**						
Increased	**T1**	27.1 (29)[1]	32.9 (27)[1]	52.5 (62)[2]	67.8 (99)[2]	<0.001 (d = 0,61)
	**T2**	28.0 (30)	23.2 (19)	33.1 (39)	39.0 (57)	
**p****		0.841 (d = 0,08)	0.088 (d = 0,51)	<0.001 (d = 0,61)	<0.001 (d = 0,69)	
Decreased	**T1**	17.8 (19)[1]	6.1 (5)[1,2]	5.9 (7)[2]	3.4 (5)[2]	<0.001 (d = 0,65)
	**T2**	10.3 (11)	13.4 (11)	9.3 (11)	17.1 (25)	
**p****		0.074 (d = 0,45)	0.003 (d = 0,79)	0.285 (d = 0,18)	<0.001 (d = 0,75)	
Remained the same	**T1**	24.3 (26)	31.7 (26)	22.0 (26)	17.8 (26)	0.116 (d = 0,19)
	**T2**	33.6 (36)	29.3 (24)	31.4 (37)	25.3 (37)	
**p****		0.086 (d = 0,51)	0.739 (d = 0,12)	0.056 (d = 0,35)	0.116 (d = 0,18)	
Does not use	**T1**	30.8 (33)[1]	29.3 (24)[1]	19.5 (23)[1,2]	11.0 (16)[2]	<0.001 (d = 0,48)
	**T2**	26.3 (31)	34.1 (28)	26.3 (31)	18.5 (27)	
**p****		0.532 (d = 0,13)	0.394 (d = 0,16)	0.074 (d = 0,38)	0.022 (d = 0,58)	
**Use of food delivery services**						
Increased	**T1**	45.8 (49)	46.3 (38)	48.3 (57)	57.5 (84)	0.201 (d = 0,11)
	**T2**	48.6 (52)	37.8 (31)	46.6 (55)	56.2 (82)	
**p****		0.631 (d = 0,12)	0.108 (d = 0,29)	0.746 (d = 0,10)	0.773 (d = 0,10)	
Decreased	**T1**	13.1 (14)	14.6 (12)	15.3 (18)	11.6 (17)	0.838 (d = 0,07)
	**T2**	13.1 (14)	17.1 (14)	11.9 (14)	12.3 (18)	
**p****		1.000 (0)	0.617 (d = 0,09)	0.414 (d = 0,10)	0.853 (d = 0,08)	
Remained the same	**T1**	21.5 (23)	19.5 (16)	20.3 (24)	15.8 (23)	0.663

	**T2**	23.4 (25)	31.7 (26)	28.0 (33)	18.5 (27)	(d = 0,07)
**p****		0.739 (d = 0,02)	0.059 (d = 0,41)	0.139 (d = 0,22)	0.480 (d = 0,15)	
Does not use	**T1**	19.6 (21)	19.5 (16)	16.1 (19)	15.1 (22)	0.726 (d = 0,06)
	**T2**	15.0 (16)	13.4 (11)	13.6 (16)	13.0 (19)	
**p****		0.197 (d = 0,18)	0.166 (d = 0,21)	0.467 (d = 0,08)	0.439 (d = 0,06)	
**Home cooking**						
Increased	**T1**	66.4 (71)	58.5 (48)	69.5 (82)	67.8 (99)	0.498 (d = 0,1)
	**T2**	40.2 (43)	40.2 (33)	44.1 (52)	41.1 (60)	
**p****		<0.001 (d = 0,47)	0.016 (d = 0,45)	<0.001 (d = 0,62)	<0.001 (d = 0,69)	
Decreased	**T1**	5.6 (6)	6.1 (5)	3.4 (4)	8.9 (13)	0.319 (d = 0,15)
	**T2**	20.6 (22)	7.3 (6)	11.0 (13)	24.7 (36)	
**p****		0.002 (d = 0,69)	0.739	0.020 (d = 0,57))	<0.001 (d = 0,45)	
Remained the same	**T1**	28.0 (30)	35.4 (29)	26.3 (31)	22.6 (33)	0.220 (d = 0,12)
	**T2**	39.3 (42)	51.2 (42)	44.9 (53)	32.2 (47)	
**p****		0.040 (d = 0,54)	0.016 (d = 61)	<0.001 (d = 0,65)	0.048 (d = 0,51)	

Generalized Estimating Equations *between-group (different numbers indicate significant changes) and **within-group analyses

Table S3 – Types of meals consumed of study participants at T0, T1, and T2 for each weight variation group

	T	**Lost (n=107) % (n)**	**Maintained (n=82) % (n)**	**Gained up to 2.4kg (n=118) % (n)**	**Gained ≥ 2.5kg (n=146) % (n)**	**p***
Breakfast						
	**T0**	86.9 (93)	90.2 (74)	91.5 (108)	90.4 (132)	
	**T1**	84.1 (90)	91.5 (75)	87.3 (103)	84.2 (123)	0.407 (d = 0,08)
	**T2**	85.0 (91)	92.7 (76)	93.2 (110)	87.0 (127)	
**p****		0.627 (d = 0,11)	0.779 (d = 0,10)	0.101 (d = 0,21)	0.157 (d = 0,18)	
Morning snack						
	**T0**	44.9 (48)[a]	53.7 (44)[a]	47.5 (56)[a]	49.3 (72)[a]	
	**T1**	30.8 (33)[b]	37.8 (31)[b]	39.0 (46)[ab]	34.2 (50)[b]	0.588 (d = 0,12)
	**T2**	30.8 (33)[b]	32.9 (27)[b]	35.6 (42)[b]	27.4 (40)[b]	
**p****		0.019 (d = 0,62)	0.001 (d = 0,59)	0.043 (d = 0,68)	<0.001 (d = 0,71)	
Lunch						

	**T0**	99.1 (106)	96.3 (79)	98.3 (116)	99.3 (145)	
	**T1**	98.1 (105)	97.6 (80)	98.3 (116)	97.3 (142)	0.938 (d = 0,08)
	**T2**	99.1 (106)	100 (82)	98.3 (116)	97.9 (143)	
**p****		0.717 (0,06)	0.174 (d= 0,12)	1.000 (d = 0)	0.311 (d = 0,11)	
Afternoon snack						
	**T0**	83.2 (89)	85.4 (70)	82.2 (97)	79.5 (116)	
	**T1**	84.1 (90)	82.9 (68)	82.2 (97)	84.2 (123)	0.969 (d = 0,09)
	**T2**	84.1 (90)	85.4 (70)	89.0 (105)	82.9 (121)	
**p****		0.967 (d = 0,08)	0.801 (d = 0,08)	0.110 (d = 0,12)	0.386 (d = 0,13)	
Dinner						
	**T0**	75.7 (81)	85.4 (70)	82.2 (97)	75.3 (110)	
	**T1**	76.6 (82)	82.9 (68)	81.4 (96)	80.8 (118)	0.711 (d = 0,12)
	**T2**	76.6 (82)	82.9 (68)	76.3 (90)	79.5 (116)	
**p****		0.961 (d = 0,1)	0.790 (d = 0,2)	0.203 (d = 0,1)	0.197 (d = 0,12)	
Evening snack						
	**T0**	24.3 (26)	32.9 (27)	18.6 (22)	26.7 (39)[a]	
	**T1**	22.4 (24)	29.3 (24)	28 (33)	37.7 (55)[b]	0.064 (d = 0,36)
	**T2**	24.3 (26)	28.0 (23)	21.2 (25)	26.7 (39)[a]	
**p****		0.882 (d = 0,1)	0.674 (d = 0,18)	0.088 (d = 0,51)	0.030 (d = 0,49)	
Other meals						
	**T0**	13.1 (14)	7.3 (6)	7.6 (9)	5.5 (8)[a]	
	**T1**	13.1 (14)	8.5 (7)	9.3 (11)	17.1 (25)[b]	0.163
	**T2**	9.3 (10)	6.1 (5)	8.5 (10)	13.7 (20)[ab]	
**p****		0.449 (d = 0,26)	0.607 (d = 0,13)	0.819 (d = 0,07)	0.002 (d = 0,60)	

Generalized Estimating Equations *between-group and **within-group analyses (different letters indicate significant changes)

Table S4 - Frequency of food consumption of study participants at T0, T1, and T2 for each weight variation group

	T	Lost (n=107)	Maintained (n=82)	Gained up to 2.4kg (n=118)	Gained ≥ 2.5kg (n=146)	p
Legumes (times/week)	**T0**	7[a] (5 – 10)	7 (5 – 10)	7 (5 – 10)	7 (5 – 10)	
	**T1**	7[ab] (5 – 10)	7 (5 – 10)	7 (5 – 10)	7 (5 – 10)	0.904 (d = 0,05)
	**T2**	7[b] (2.5 – 7)	7 (5 – 7)	7 (5 – 10)	7 (2.5 – 7.7)	
**p****		0.046* (d = 0,52)	0.228 (d = 0,11)	0.095 (d = 0,19)	0.140 (d = 0,10)	
Vegetables (times/week)	**T0**	7 (5 – 10)	7 (5 – 10)	7 (5 – 10)	7[a] (5 – 10)	
	**T1**	7 (7 – 10)	7 (5 – 10)	7 (5 – 10)	7[ab] (5 – 10)	0.467 (d = 0,09)

	**T2**	7 (5 – 10)	7 (5 – 10)	7 (5 – 10)	7[b] (5 – 10)	
**p****		0.338 (d = 0,21)	0.210 (d = 0,12	0.514 (d = 0,08)	0.033 (d = 0,34)	
Fresh Fruits (times/week)	**T0**	7 (5 – 10)	7 (5 – 10)	7 (5 – 10)	7 (2.5 – 10)	0.007 (d = 0,39)
	**T1**	7 (5 – 10)[1]	7 (5 – 10)[1,2]	7 (4.3 – 10)[1]	6 (2.5 – 10)[2]	
	**T2**	7 (5 – 10)	7 (5 – 10)	7 (2.5 – 10)	7 (2.5 – 10)	
**p****		0.236 (d = 0,10)	0.455 (d = 0,12)	0.177 (d = 0,08)	0.282 (d = 0,12)	
Cereals (times/week)	**T0**	7 (7 – 10)	7 (7 – 10)	7 (7 – 10)	7 (5 – 10)	0.820 (d = 0,07)
	**T1**	7 (7 – 10)	7 (7 – 10)	7 (7 – 10)	7 (5 – 10)	
	**T2**	7 (5 – 10)	7 (5 – 10)	7 (5 – 10)	7 (5 – 10)	
**p****		0.164 (d = 0,12)	0.257 (d = 0,11)	0.215 (d = 0,11)	0.433 (d = 0,09)	
Meats (times/week)	**T0**	7 (5 – 10)	7 (5 – 10)	7 (5 – 10)	7 (5 – 10)	0.147 (d = 0,12)
	**T1**	7 (5 – 10)	7 (4.3 – 10)	7 (5 – 10)	7 (5 – 10)	
	**T2**	7 (5 – 10)	7 (5 – 10)	7 (5 – 10)	7 (5 – 10)	
**p****		0.327 (d = 0,12)	0.064 (d = 0,31)	0.429 (d = 0,15)	0.369 (d = 0,08)	
Milk and dairy products (times/week)	**T0**	7 (5 – 10)	7 (4.3 – 10)	7 (5 – 10)	7 (4.3 – 10)	0.938 (d = 0,08)
	**T1**	7 (2.5 – 10)	7 (2.5 – 10)	7 (5 – 10)	7 (5 – 10)	
	**T2**	7 (2.5 – 7)	7 (2.5 – 10)	7 (2.5 – 10)	7 (2.5 – 10)	
**p****		0.069 (d = 0,48)	0.841 (d = 0,05)	0.726 (d = 0,07)	0.196 (d = 0,25)	
Bakery products (times/week)	**T0**	7 (5 – 10)	7 (5 – 10)	7 (7 – 10)	7[ab] (5 – 10)	0.272 (d = 0,15)
	**T1**	7 (5 – 10)	7 (5 – 10)	7 (5 – 10)	7[a] (5 – 10)	
	**T2**	7 (5 – 10)	7 (5 – 10)	7 (5 – 10)	7[b] (5 – 10)	
**p****		0.786 (d = 0,10)	0.387 (d = 0,15)	0.121 (d = 0,14)	0.015 (d = 0,75)	
Hamburgers or canned products (times/week)	**T0**	1.0 (0.5 – 2.5)	0.5 (0.5 – 1.4)	0.5 (0.5 – 1)	1.0[a] (0.5 – 2.5)	0.001 (d = 0,74)
	**T1**	0.5 (0.5 – 2.5)[1]	0.5 (0.5 – 1)[1]	1.0 (0.5 – 2.5)[1,2]	2.5[b] (0.5 – 5)[2]	
	**T2**	1.0 (0.5 – 2.5)	0.5 (0.5 – 1)	1.0 (0.5 – 2.5)	1.0[a] (0.5 – 2.5)	
**p****		0.303 (d = 0,15)	0.493 (d = 0,14)	0.117 (d = 0,05)	0.003 (d = 0,59)	
Sugary drinks (times/week)	**T0**	1.0 (0.5 – 2.5)	0.5 (0.5 – 2.5)	0.5 (0.5 – 2.5)	1.0[ab] (0.5 – 5)	0.011 (d = 0,49)
	**T1**	0.5 (0.5 – 2.5)[1]	0.5 (0.5 – 2.5)[1]	0.5 (0.5 – 2.5)[1]	1[a] (0.5 – 5)[2]	
	**T2**	0.5 (0.5 – 2.5)	0.5 (0.5 – 2.5)	0.5 (0.5 – 2.5)	0.5[b] (0.5 – 2.5)	

**p****		0.576 (d = 0,09)	0.777 (d = 0,1)	0.547 (d = 0,09)	0.011* (d = 0,61)	
Instant meals and snacks (times/week)	**T0**	0.5 (0.5 – 1)	0.5 (0.5 – 1)	0.5 (0.5 – 1)	0.5[a] (0.5 – 1.3)	<0.001 (d = 0,52)
	**T1**	0.5 (0.5 – 1)[1]	0.5 (0 – 1)[1]	0.5 (0.5 – 1)[1,2]	1[b] (0.5 – 2.5)[2]	
	**T2**	0.5 (0.5 – 1)	0.5 (0 – 0.5)	0.5 (0.5 – 1)	0.5[a] (0.5 – 1)	
**p****		0.690 (d = 0,05)	0.074 (d = 0,54)	0.291 (d = 0,12)	0.001 (d = 0,38)	
Candies (times/week)	**T0**	1.0[a] (0.5 – 2.5)	1.0 (0.5 – 2.5)	2.5[a] (1 – 5)	2.5[a] (1 – 5)	0.002 (d = 0,60)
	**T1**	1.0[ab] (0.5 – 5)[1]	2.5 (0.5 – 5)[1]	2.5[b] (1 – 7)[1,2]	5.0[b] (1 – 7)[2]	
	**T2**	2.5[b] (1 – 5)	1.0 (0.5 – 5)	2.5[a] (0.5 – 5)	2.5[a] (1 – 5)	
**p****		0.023 (d = 0,35)	0.087 (d = 0,27)	0.006 (d = 0,49)	<0.001 (d = 0,53)	
Fast food (times/week)	**T0**	1 (0.5 – 1)	1 (0.5 – 1)	1 (0.5 – 1)	1[a] (0.5 – 2.5)	<0.001 (d = 0,45)
	**T1**	1 (0.5 – 1)[1]	1 (0.5 – 1)[1]	1 (0.5 – 2.5)[1]	1[b] (1 – 2.5)[2]	
	**T2**	1 (0.5 – 1)	1 (0.5 – 1)	1 (0.5 – 1)	1[a] (0.5 – 2.5)	
**p****		0.611 (d = 0,12)	0.952 (d = 0,08)	0.218 (d = 0,16)	0.001 (d = 0,46)	

Generalized Estimating Equations *between-group (different numbers indicate significant changes) and **within-group analyses (different letters indicate significant changes).

Table S5 - Eating behavior and perceived stress of study participants at T1 and T2 for each weight variation group

	T	**Lost (n=107)**	**Maintained (n=82)**	**Gained up to 2.4kg (n=118)**	**Gained ≥ 2.5kg (n=146)**	**p***
Cognitive restriction	**T1**	44.4 (27.7 -61.1)	38.8 (16.6 – 54.1)	44.4 (27.7 – 61.1)	44.4 (33.3 - 61.1)	0.062 (d = 0,36)
	**T2**	38.8 (22.2 - 61.1)	38.8 (22.2 – 55.5)	38.8 (27.7 – 61.1)	44.4 (27.7 - 61.1)	
**p****		0.251 (d = 0,18)	0.617 (d = 0,08)	0.136 (d = 0,12)	0.635 (d = 0,08)	
Uncontrolled eating	**T1**	25.9 (14.8 – 40.7)[1]	20.3 (8.3 - 36.1)[1]	25.9 (16.6 – 44.4)	40.7 (22.2 - 55.5)[2]	<0.001 (d = 0,71)
	**T2**	25.9 (11.1 – 44.4)	22.2 (7.4 - 37.0)	25.9 (17.6 - 40.7)	33.3 (17.6 - 52.7)	
**p****		0.583 (d = 0,07)	0.900 (d = 0,10)	0.915 (d = 0,07)	0.007 (d = 0,68)	
Emotional eating	**T1**	27.7 (5.5 – 50.0)[1]	27.7 (5.5 – 38.8)[1]	27.7 (11.1 – 50.0)[1]	50.0 (22.2 - 72.2)[2]	<0.001 (d = 0,60)
	**T2**	22.2	22.2	33.3	44.4	

		(5.5 – 50.0)	(4.1 - 50.0)	(9.7 - 51.4)	(22.2 - 66.6)	
**p****		0.036 (d = 0,71)	0.819 (d = 0,12)	0.829 (d = 0,07)	0.043 (d = 0,52)	
Perceived stress	**T1**	23.0 (19.0 – 27.0)[1,2]	20.0 (15.2 – 27.7)[1]	22.0 (16.0 – 27.5)[1]	25.0 (21 – 30.0)[2]	0.004 (d = 0,51)
	**T2**	26.0 (23.0 – 28.0)	25.0 (22.0 – 29.0)	25.0 (22.0 – 29.0)	26.0 (22.0 –29.0)	
**p****		0.002 (d = 0,58)	<0.001 (d = 0,47)	<0.001 (d = 0,52)	0.857 (d = 0,13)	

Generalized Estimating Equations *between-group (different numbers indicate significant changes) and **within-group analyses

Table S6 – Classification of weight variation of participants in T1, according to participation in the second phase (T2) of the study

	**Lost % (n)**	**Maintained % (n)**	**Gained up to 2.4kg % (n)**	**Gained ≥ 2.5kg % (n)**	**p**
Responded to the 2nd phase (n=453)	23.6 (107)	18.1 (82)	26.0 (118)	32.2 (146)	0.105 (d = 0,08)
Did not respond to the 2nd phase (n=885)	25.9 (229)	15.9 (141)	21.4 (189)	36.8 (326)	

Pearson's Chi-Square Test
